# Bulk Heterojunction Solar Cell with Nitrogen-Doped Carbon Nanotubes in the Active Layer: Effect of Nanocomposite Synthesis Technique on Photovoltaic Properties

**DOI:** 10.3390/ma8052415

**Published:** 2015-05-08

**Authors:** Godfrey Keru, Patrick G. Ndungu, Genene T. Mola, Vincent O. Nyamori

**Affiliations:** 1 School of Chemistry and Physics, University of KwaZulu-Natal, Private Bag X54001, Durban 4000, South Africa; E-Mails: 212561611@stu.ukzn.ac.za (G.K.); Mola@ukzn.ac.za (G.T.M.); 2 Department of Applied Chemistry, University of Johannesburg, P.O. Box 17011, Doornfontein, Johannesburg 2028, South Africa; E-Mail: pndungu@uj.ac.za

**Keywords:** polythiophene, nitrogen-doped carbon nanotubes, nanocomposites, photovoltaic properties

## Abstract

Nanocomposites of poly(3-hexylthiophene) (P3HT) and nitrogen-doped carbon nanotubes (N-CNTs) have been synthesized by two methods; specifically, direct solution mixing and *in situ* polymerization. The nanocomposites were characterized by means of transmission electron microscopy (TEM), scanning electron microscopy (SEM), X-ray dispersive spectroscopy, UV-Vis spectrophotometry, photoluminescence spectrophotometry (PL), Fourier transform infrared spectroscopy (FTIR), Raman spectroscopy, thermogravimetric analysis, and dispersive surface energy analysis. The nanocomposites were used in the active layer of a bulk heterojunction organic solar cell with the composition ITO/PEDOT:PSS/P3HT:N-CNTS:PCBM/LiF/Al. TEM and SEM analysis showed that the polymer successfully wrapped the N-CNTs. FTIR results indicated good π-π interaction within the nanocomposite synthesized by *in situ* polymerization as opposed to samples made by direct solution mixing. Dispersive surface energies of the N-CNTs and nanocomposites supported the fact that polymer covered the N-CNTs well. J-V analysis show that good devices were formed from the two nanocomposites, however, the *in situ* polymerization nanocomposite showed better photovoltaic characteristics.

## 1. Introduction

The electrical conductivity of a linear chain organic polymer (polyacetylene) was first discovered by Shirikawa *et al.* in 1977 [1]. Later, a number of other organic molecules particularly polythiophene groups emerged as alternative conducting polymers (CPs) with potential applications in the area of opto-electronic devices. The advantages of CPs over inorganic semi-conductors in opto-electronic applications include the ease of processability, tuneable properties of the molecules, flexibility, and being light weight [2]. CPs consist of alternating single and double bonds; the π-electrons in their double bonds are mobile due to overlap of π-orbitals [3]. Electronic properties of CPs can be tuned during synthesis and they also have good magnetic and optical properties [4]. Of late, CPs have been considered as one of the best alternatives for the production of solar cells. This realization stems from the fact that solar cells fabricated by use of inorganic materials can be expensive and also the processes that are utilized during their manufacture can be extensively energy intensive [5]. For CPs to function well in solar cells they should possess the following characteristics: soluble in common organic solvents, can form a thin film on substrates, low band-gap to enhance absorption, partially miscible with electron acceptors, a good hole conductor, and chemically stable in ambient conditions [3]. Polythiophenes not only have several of the characteristics named above, but also have efficient electronic conjugation, as well as synthetic versatility [3]. Polythiophenes are made by linking thiophene rings, which are insoluble, and then improving their solubility by introducing alkyl chains, *i.e.*, hexyl and octyl groups. Polythiophenes have been studied for various applications, which include organic field effect transistors [6], solar cells [7], sensors, and light emitting diodes [8].

Although CPs have unique properties their applications in various fields are hampered by their poor environmental stability and mechanical strength. This problem can be overcome by introducing different fillers within the CPs to form nanocomposites [9,10]. Among the different fillers available, carbon nanotubes (CNT) have attracted a great deal of interest due to their unique structural, electrical, and mechanical properties [11]. Further enhancement of these unique properties can be achieved by doping CNTs with boron or nitrogen to form boron- and nitrogen-doped CNTs (B-CNTs or N-CNTs). Nitrogen-doping creates defects on the walls of CNTs that improves the ability of the surfaces to undergo various covalent chemistries, provide good anchoring sites for nanoparticles, introduces a variety of functional groups, and more importantly, it also improves the electrical conductivity of CNTs [12]. For example, Panchakarla *et al.* [13] reported higher electrical conductivity for N-CNTs than for pristine CNTs. CNTs/N-CNTs in polymer nanocomposites enhance Young’s modulus, the tensile strength, and electrical conductivity [14]. Additionally, the incorporation of CNTs can be an effective route to synthesize low density, high performance thermoelectric materials [15]. Nanocomposites of CNTs coated with CPs have found use in organic field emission devices [16], light emitting diodes [8,17], electronic devices and sensors [18], and organic solar cells (OSC) [19]. However, effective utilization of CNTs in the polymer nanocomposites strongly depends on the dispersion. For example, poorly dispersed CNTs in the active layer of OSC can act as recombination sites and can also lead to short-circuiting [20].

OSCs have gained a great deal of attention in recent years due to the high expectation of producing relatively cheap devices for converting solar energy directly to electricity [10]. Some of the advantages of OSCs over inorganic solar cells include low-cost manufacturing, high-throughput production, and high flexibility, and, therefore, OSCs can be cast on flexible substrates or on curved surfaces [19]. Although CNTs have many advantages when well dispersed in the polymer matrix, the performance of OSCs with CNTs in the active layer have continued to perform poorly when compared to polymer/fullerene systems, e.g., poly(3-hexylthiophine) (P3HT) and [6,6]-phenyl-C_61_-butyric acid methyl ester (PCBM). This has been attributed to short-circuiting as a result of a mixture of semi-conducting and metallic CNTs [21], and filamentary short-circuiting due to CNTs extending outside the active layer [22]. Poor performance could also be due to unbalanced charge mobility for a device with CNTs, as one charge carrier will be transferred very fast while the other is subjected to hopping in disordered organic materials [23].

Jun *et al.* [24] fabricated a solar cell with CNTs functionalized with alkyl-amides in an active layer of P3HT:PCBM. The efficiency of this device increased by 30% from 3.2% to 4.4% compared with a device without CNTs. They attributed this increase to wide band absorption, high charge carrier mobility and improved dispersion in the polymer matrix. Kalita *et al.* [25] used plasma oxygen-functionalized CNTs in the active layer of P3HT:PCBM and reported an 81.8% efficiency increase from 1.21% to 2.2% compared with a device without CNTs. They attributed this increase to improved hole mobility and increased surface area for excitons dissociation.

In this paper we compare the effect of synthesis technique for nanocomposites on their photovoltaic properties. The two techniques compared are: oxidative *in situ* polymerization, and direct solution mixing of P3HT and N-CNTs. We also report on a unique characterization technique whereby dispersive surface energy was used to determine how effective the polymer wrapped/covered the walls of N-CNTs. Finally, the results on the use of nanocomposites in the active layer of organic solar cells (OSC) is presented and discussed.

## 2. Experimental Section

### 2.1. Materials

Chemicals used in this study were of analytical grade and were used as received unless stated otherwise. Anhydrous ferric chloride (99%), [6,6]-phenyl-C_61_-butyric acid methyl ester (PCBM) (98%), regioregular poly (3-hexylthiophene-2,5-diely (99%), and 3-hexylthiophene (99%) were purchased from Sigma Aldrich (St. Louis, MO, USA) while, chloroform (99%) was sourced from an alternative supplier (Merck Chemicals, S.A). Indium tin oxide (ITO) coated glasses slide was purchased from Merck, Germany. Chloroform was dried before being used.

### 2.2. Synthesis of Nitrogen-Doped CNTs and Nanocomposites

N-CNTs were synthesized in our laboratory by a chemical vapor deposition floating catalyst method as reported elsewhere [26]. Briefly, 2.5 wt% of 4-pyridinyl-4-aminomethylidinephenylferrocene catalyst was dissolved in acetonitrile solvent to make 100 wt% solution. This was followed by pyrolysis at 850 °C. The crude N-CNTs obtained were purified by, firstly, calcining at 400 °C in air to remove amorphous carbon, and then refluxing with 6 M HNO_3_ for 24 h at 80 °C to remove any iron residue used as catalyst during the N-CNTs synthesis.

The nanocomposites were synthesized by the means of two techniques, namely, oxidative *in situ* polymerization and direct solution mixing. For oxidative *in situ* polymerization of 3-hexylthiophene monomers on the walls of N-CNTs, this was achieved by use of a similar method as reported by Karim [27]. In brief, 1 wt% (of the weight of 3HT monomers) of N-CNTs (6.8 mg) was weighed, 50 mL of dry chloroform was added and the mixture was placed in a two-necked round-bottomed flask with a stirrer. The mixture was sonicated for 1 h to disperse the N-CNTs. Thereafter, 0.648 g (4 mmol) of anhydrous ferric chloride in 50 mL of dry chloroform was added to the above dispersion and further sonicated for 30 min. Then 673.2 mg (2 mmol) of 3-hexylthiophene monomers in 25 mL of dry chloroform solution was placed in a pressure-equalized funnel and added dropwise to the above mixture with constant stirring. Stirring continued under the same conditions for the next 24 h. The nanocomposite was precipitated with methanol; vacuum filtered, washed with methanol, 0.1 M HCl, deionized water, acetone, and then eventually vacuum dried for 24 h at room temperature.

The direct solution mixing method to synthesize the nanocomposite was adapted from a method reported by Lee *et al.* [28]. In brief, P3HT was dissolved in dry chloroform to make 20 mg·mL^−1^ solution, and 1 wt% of N-CNT (of the weight of P3HT) were added to the solution of P3HT in chloroform. The mixture was sonicated for 1 h then stirred for 12 h in the dark to protect it from light. This solution was spin coated directly on ITO glass substrate.

The active layer for the solar cell device was prepared by mixing the N-CNTs/P3HT nanocomposite with [6,6]-phenyl-C_61_-butyric acid methyl ester (PCBM) at a ratio of 1:0.8 by mass to make 20 mg·mL^−1^ solution in chloroform. The mixture was sonicated for two hours before spin-coating onto ITO coated glass substrates.

### 2.3. Characterization of the Nanocomposites

The morphology and structure of the nanocomposites was characterized by; transmission electron microscopy (TEM), (JEOL JEM 1010, JEOL Ltd., Tokyo, Japan) at 200 kV. The nanocomposite samples were dispersed in ethanol by sonication before being deposited on carbon-coated copper grids. A scanning electron microscope (Carl Zeiss ultra plus field emission electron microscopy (FEGSEM), Carl Zeiss, Cambridge, UK) was used at 5 kV accelerating voltage. Samples were placed on aluminum stubs by using carbon tape.

Raman spectra of N-CNTs and the nanocomposites were recorded with a DeltaNu Advantage 532TM Raman spectrometer (DeltaNu, Vancouver, BC, Canada). The excitation source was a Nd:YAG solid state crystal class 3b diode laser at 532 nm excitation wavelength. Nuspec TM software was used to capture generated spectra. Thermogravimetric analysis (TGA) was performed with a TA Instruments Q series^TM^ thermal analyzer DSC/TGA (Q600). The P3HT, nanocomposites and N-CNTs were heated at a rate of 10 °C·min^−1^ under an air flow rate of 50 mL·min^−1^ and the data was captured and analyzed using the TA Instrument Universal Analysis 2000 software (TA Instrument, New Castle, DE, USA).

An inverse gas chromatography (IGC) surface energy analyzer (SEA) (Cirrus, Dublin, Ireland) was used to determine the surface energy properties of N-CNTs and the nanocomposites. About 30 mg of the sample was packed in an IGC salinized glass column of 300 mm length and 4 mm internal diameter. The column was filled with salinized glass wool on both ends of the sample until it was well packed. Cirrus control software was used to control the analysis and Cirrus plus software was used for data analysis. FTIR spectra were recorded with KBr pellets on a Perkin-Elmer Spectrum 1 FTIR spectrometer (PerkinElmer, Waltham, MA, USA) equipped with spectrum Rx software. Photoluminescence spectra were obtained with a Perkin Elmer Spectro Fluorimeter equipped with FL Winlab software at an excitation wavelength of 298 nm in chloroform solution. UV-Vis spectra were recorded in a chloroform solution with a Perkin Elmer Lamba 35 dual-beam UV-Vis spectrophotometer and data analyzed with FL Winlab software. Samples for the Photoluminescence and UV-Vis were prepared using a modified method as reported by Goutam *et al.* [14], briefly, 10 mg of P3HT and nanocomposites were dissolved in dry chloroform to make 100 mL solutions. Necessary equivalent dilutions were made using micropipette to record spectra (Cleveland, QC, Canada).

### 2.4. Device Preparation

Devices were prepared in ambient conditions on ITO-coated glass substrates with a shunt resistance of 15 Ω. Half of the ITO coat was etched with a mixture of water, HCl and HNO_3_ in the ratio of 12:12:1 by volume, respectively, and then placed in deionized water. The etched substrate was thereafter cleaned by sonication for 10 min each with separate solution of detergent, distilled water, acetone, and finally with isopropanol. Thereafter, the substrate was dried at 50 °C for 10 min. The hole transport layer poly(3,4-ethylenedioxythiophene): poly(styrenesulfonate) (PEDOT:PSS) was spin-coated on the clean substrate at 3000 rpm for 50 s, and then annealed for 10 min at 120 °C. The active layer, a mixture of P3HT/N-CNTs:PCBM was spin-coated at 1500 rpm for 30 s, and then annealed for 20 min at 120 °C. Before vacuum evaporation of the counter electrode, 0.6 nm of lithium fluoride (LiF) was evaporated on top to serve as a hole-blocking layer. A 60 nm counter electrode consisting of Al metal was thermally evaporated at 2.22 × 10^−7^ mbar in an HHV Auto 306 vacuum evaporator equipped with INFICON SQM-160 thin film deposition thickness and rate monitor. Current-voltage characterization was determined by using a standard solar simulator model #SS50AAA (Pet Photoemission Tech. Inc., Camarillo, CA, USA), with a Keithley 2420 source meter.

## 3. Results and Discussion

**Scheme 1:**
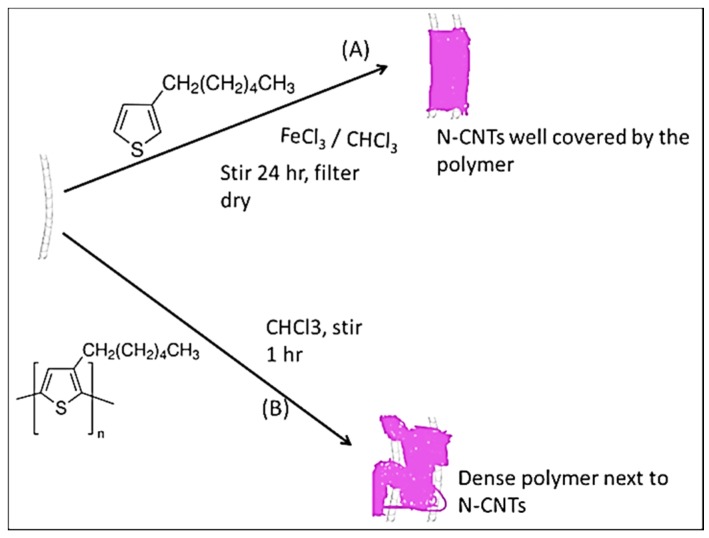
Synthesis of the nanocomposites (**A**) *in situ* polymerization; and (**B**) direct mixing.

Scheme 1 illustrates how the synthesis of the nanocomposites was achieved. In the *in situ* polymerization technique, monomers were polymerized directly on the surface of the N-CNTs. However, in the direct solution mixing, a solution of N-CNTs was mixed with a solution of the polymer.

### 3.1. Morphology and Structure of the Nanocomposite

**Figure 1:**
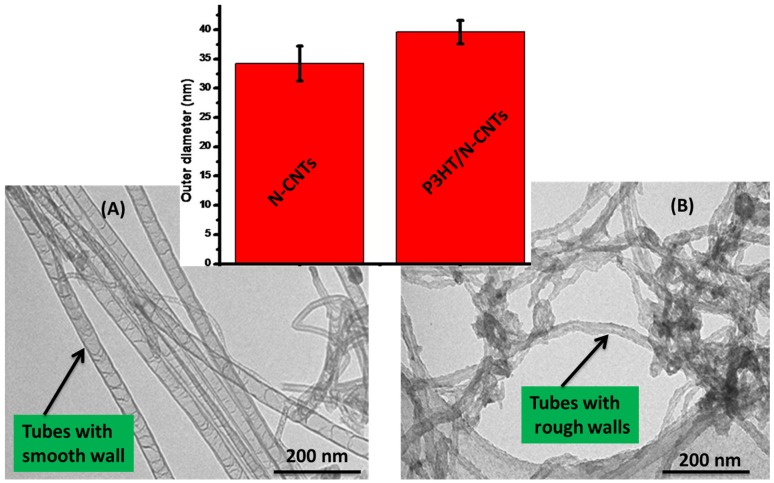
TEM images of (**A**) purified N-CNTs; and (**B**) N-CNT/P3HT nanocomposite synthesized by *in situ* polymerization (inset comparison of outer diameters).

Figure 1 shows the structure of the N-CNTs before and after formation of the nanocomposites with poly(3-hexylthiophene). From the TEM images it was observed that the polymer coated the surface of the N-CNTs. From the bamboo structures observed, it can be deduced that the tubular inner part consist mainly of N-CNTs and the coated surface is conducting P3HT. The smooth surfaces of the N-CNTs (Figure 1A) became rough after they were covered by the polymer (Figure 1B).

**Figure 2:**
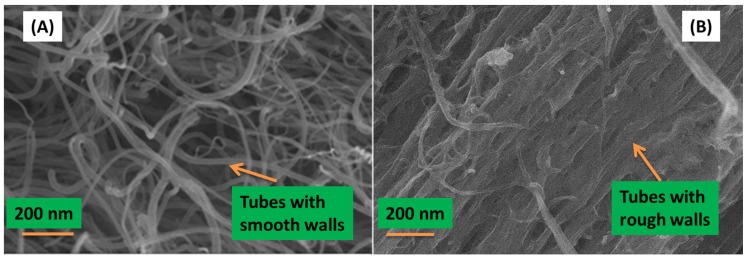
Morphology of (**A**) N-CNTs; and (**B**) nanocomposite of N-CNTs/P3HT synthesized by *in situ* polymerization.

Figure 2 shows the morphology of the N-CNTs and nanocomposite synthesized by *in situ* polymerization as observed in SEM.

From Figure 2A, the N-CNTs appear as an entangled mat of tubular structures with smooth surfaces which is a characteristic of carbon-based nanotubes. However, in Figure 2B a few thick tubular structures with rough surfaces and agglomerated mat-like structures were observed, and this is indicative of the polymer wrapping onto the nanotubes to form a nanocomposite. EDX, which was coupled with FEGSEM, provided further evidence of the nanocomposite elemental composition, which consisted of carbon, sulfur and oxygen. Oxygen observed in the nanocomposite could be due to the introduction of oxygenated groups during acid purification and functionalization of N-CNTs. The at% of carbon increased with the formation of the nanocomposite as compared to that in polymer. Karim [27] reported similar results when they synthesized P3HT/MWCNTs nanocomposites by *in situ* polymerization.

Further evidence of the polymer wrapping the N-CNTs was obtained by measuring the outer diameters of the N-CNTs and nanocomposite from their TEM images, (inset Figure 1). This was determined from not less than 50 TEM images and over 200 tubes per sample. The diameters were observed to increase with formation of the nanocomposites. An increase of ≈15.9% was observed; a good indication that the N-CNTs were wrapped by the polymer.

**Figure 3:**
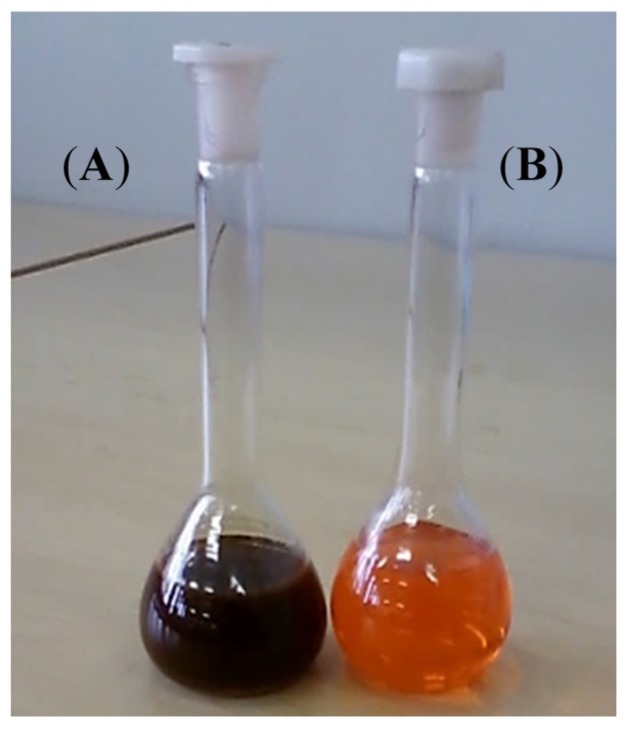
Color of P3HT in chloroform solution (**A**) after formation of nanocomposite and (**B**) pristine P3HT under white light.

Figure 3 further confirms formation of a nanocomposite whereby, the brown color of pristine P3HT changed to dark brown. Our observations concur with what is reported elsewhere in the literature [29].

### 3.2. Vibrational and Spectral Characteristics of P3HT and the Nanocomposite

**Figure 4:**
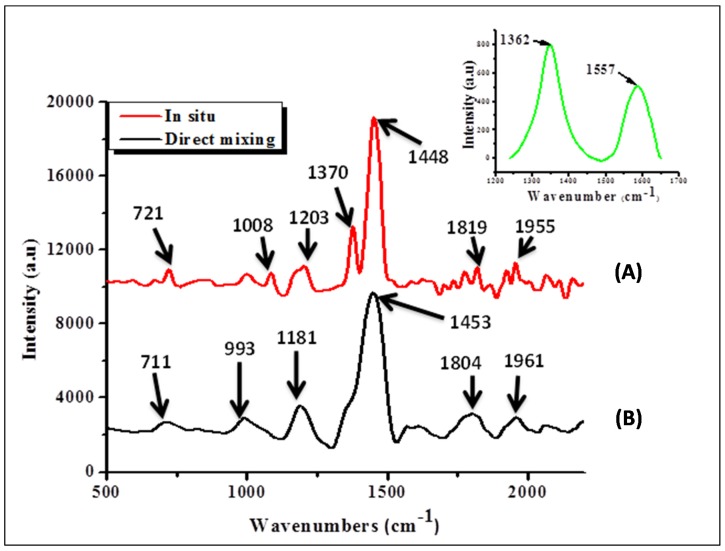
Raman spectroscopy results of nanocomposites, (A) direct mixing and (B) *in situ* polymerization (inset position of D-band and G-band for N-CNTs).

Interaction between P3HT and N-CNTs in the two nanocomposites was assessed by means of Raman spectroscopy. Figure 4 shows Raman vibration peaks of the nanocomposites.

For the N-CNTs (Figure 4 inset) the peak at 1593 nm represents the G-band, which originates from the Raman E_2g_ mode while the one at 1356 cm^−1^ is the disorder-induced band. The I_D_/I_G_ ratio for N-CNTs was 1.55, which was an indication of high disorder due to nitrogen-doping. Both peaks were consequently absent in the nanocomposites. The observed peaks for both nanocomposites were almost in similar position and can be assigned as follows; peak at 704–719 cm^−1^ is the C–S–C ring deformation for thiophene rings while that at around 1198 cm^−1^ is the C–C symmetric stretching and C–H bending vibrations. The peak in the range of 1373–1377 cm^−1^ is the C–C stretch deformation in organic thiophene rings while that around 1438–1454 cm^−1^ is the symmetric C–C stretch deformations in alkyl chain and 1800–1850 cm^−1^ is the asymmetric C–C stretching deformation for of thiophene ring [30].

**Figure 5:**
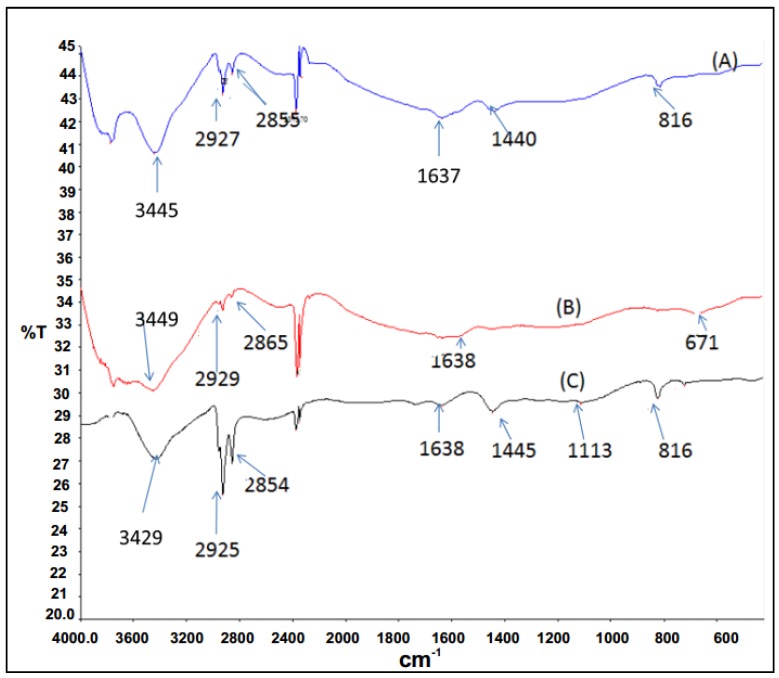
FTIR absorption frequencies for P3HT and nanocomposites (A) P3HT; (B) *in situ*; (C) direct mixing.

FTIR spectroscopy results for P3HT and P3HT/N-CNTs nanocomposites synthesized by both techniques are presented in Figure 5. P3HT shows a peaks at 2925 and 2844 cm^−1^ assigned to C–H stretching vibrations, the peak at 1645 cm^−1^ is assigned to the aryl substituted C=C of the thiophene ring, the peak at 1440 cm^−1^ is attributed to the vibrational stretch of the thiophene ring and peaks between 900 and 670 cm^−1^ are due to the C–H out-of-plane deformation of thiophene [30].

The nanocomposites synthesized by direct solution mixing gave almost all the peaks as for P3HT which was an indication of poor interaction between the polymer and N-CNTs. However, for the nanocomposite synthesized by *in situ* polymerization the C–H stretch vibration peak shifted slightly to higher wavenumbers from 2925 to 2929 cm^−1^. A slight shift to longer wavenumbers can be attributed to CH-π interaction between N-CNTs and P3HT [31]. Additionally, the peak at 1440 cm^−1^ was not observed for this nanocomposite, which was an indication that stretching vibration of thiophene ring was interfered with and also, evidence of π-π interaction between N-CNTs and the thiophene rings of P3HT [31].

**Figure 6:**
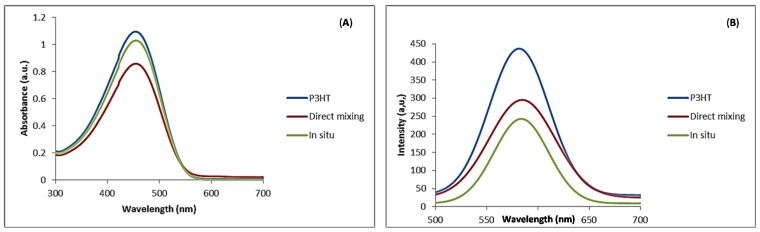
(**A**) Uv-Vis absorption spectra; (**B**) photoluminescence emission for P3HT and P3HT/N-CNTs nanocomposites in chloroform solution.

UV-visible absorption spectra of pristine P3HT and the nanocomposites in chloroform solution are presented in Figure 6A. The absorption maximum (λ_max_) for P3HT was observed at 442 nm, an indication of extensive π-conjugation [14]. The absorption peak for P3HT that we observed compared well with values reported in literature [27]. From the figure it was noted that N-CNTs did not make significant contribution to the spectra but a small red shift was noted to λ_max_ of 445 nm for the nanocomposites synthesized with both techniques. Slight red shift of λ_max_ can be attributed to an increased conjugation length of the polymer due to strong π–π interaction with N-CNTs as a result of increased organization of the polymer chains on the nanotube surface [32].

Photoluminescence (PL) spectra of P3HT and the nanocomposites are shown in Figure 6B. The emission peak of P3HT was observed at 581 nm and those of the nanocomposites were slightly red shifted to 585 nm, but the intensity of the emission peak for P3HT was higher than that of nanocomposites. This was attributed to quenching as a result of charge transfer between N-CNTs and P3HT reducing electron-hole recombination. Quenching was high for the nanocomposite synthesized by *in situ* polymerization. This can be due to better π–π interaction between the P3HT and N-CNTs surfaces enhancing the charge transfer process. Kuila *et al.* [31] attributed PL quenching to π–π interaction between the polymer and CNTs introducing additional deactivation paths for the excited electrons.

### 3.3. Thermal Stability of the Nanocomposites

**Figure 7:**
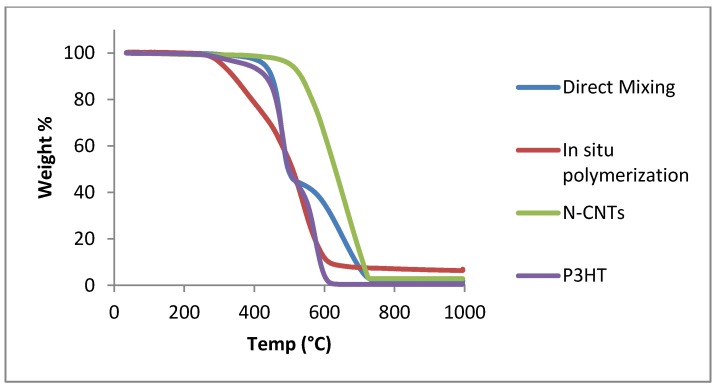
Thermogravimetric analysis of N-CNTs, P3HT and P3HT/N-CNTs nanocomposites.

The TGA thermograms for N-CNTs and the nanocomposites are presented in Figure 7. Both nanocomposites exhibit a two-step weight loss process and this could suggest that they are mixtures. The initial decomposition temperature for the nanocomposites is lower than for N-CNTs and indicates that they are less thermally stable.

The nanocomposite formed by *in situ* polymerization is the least thermally stable. The possible reason for this could be due to high reactivity of monomers polymerizing and thus, forming more polymer on the surface of N-CNTs. The high amount of residue for *in situ* polymerization nanocomposite could be due to some remnant ferric oxide initiator remaining entrapped as the nanocomposite even after washing.

### 3.4. Surface Energy Analysis of Nanocomposites

The effectiveness of the wrapping/covering of N-CNTs with the polymer was determined by comparing dispersive components of surface energy of the N-CNTs and the nanocomposites by using inverse gas chromatography equipped with a flame ionization detector. The dispersive component of surface energy ($\gamma_{s}^{d}$) can be a useful tool to examine surfaces of a solid whereby changes in surface properties can easily be detected. The $\gamma_{s}^{d}$ was obtained from the retention time (t_R_) of a given volume of a series of normal alkanes (C5–C9) at a flow rate of 10 mL·min−1 and 0.05 surface coverage. The $\gamma_{s}^{d}$ was calculated from the slope of a straight line drawn from RTlnV_N_ against the number of carbon atoms in the n-alkanes by using the Doris and Gray method at peak maximum time [33]. R is the gas constant, T is the absolute column temperature and *V*_N_ is the net retention volume of non-polar probes as well as polar probes and can be calculated from Equation (1):

(1) $$V_{\text{N}}=Fj(t_{R}-t_{M})\left(\frac{P_{0}-P_{w}}{P_{0}}\right)\left(\frac{T_{c}}{T_{meter}}\right)$$

where *F* is the flow rate; *t*_R_ and *t*_M_ are the retention and dead times measured with a specific probe and a non-adsorbing probe (such as methane) respectively; *P*_0_ is the pressure at the flow meter; *P*_w_ is the vapor pressure of pure water at the temperature of the flow meter (Tmeter); and *T*_c_ is the column temperature [34]. For *j* it was James-Martin correction factor of gas compressibility when the column inlet (*P*_i_) and outlet (*P*_0_) pressures are different given by Equation (2):

(2) j=32[(PiP0)2−1(PiP0)3−1]

The polar probes acetone, acetonitrile, ethyl acetate and dichloromethane were used to determine the acid/base properties of N-CNTs and the nanocomposites surfaces. The $\gamma_{s}^{d}$ was determined at 100 °C for the N-CNTs and the nanocomposites by using five alkanes namely, pentane, hexane, heptane, octane and nonane. Table 1 presents the data on the $\gamma_{s}^{d}$, the acid and base constants determined from the interactions with the polar probes, and the acid base ratio.

**Table 1:** $\gamma_{s}^{d}$, acid-base constants determined from interaction with polar probes.

Sample	Surface energy (mJ·m^−2^)	Acid Constant-Ka	Base Constant-Kb	Acid-Bascity ratio	Specific (Acid-Base) Free Energy (kJ·Mol^−1^)			
					Acetone	Aceto-Nitrile	Ethyl Acetate	Dichloro-Methane
N-CNTs	49.02	0.0275	0.4975	0.0552	6.75	11.8	5.20	8.14
*In Situ* Nano-composite	56.53	0.0622	0.5738	0.1084	11.1	15.7	7.74	8.49
Direct mixing Nano-composite	46.68	0.3031	0.4446	0.6819	26.3	26.4	24.5	7.35

The $\gamma_{s}^{d}$ of N-CNTs was higher than that of the nanocomposite made by the direct mixing method and lower than those samples synthesized by *in situ* polymerization. The differences can be attributed to the slight difference in the final morphology of the nanocomposites. When comparing the two nanocomposites only, the direct mixing method reduces the dispersive component of the surface energy by either effectively wrapping CNT bundles, leaving fewer exposed CNT tips, or through a combination of both factors. The *in situ* method has a slightly larger dispersive component than the N-CNTs due to the polymerization process and the combination of ultra sound effectively de-bundling the N-CNTs. This allows for a greater amount of individual N-CNTs to be wrapped by the polymer and also, allow for greater exposure of surface groups at the tips of the N-CNTs. The specific free energy ΔG^AB^ of adsorption for acid-base specific interaction was high for bi-functional acetonitrile for both N-CNTs and the nanocomposites showing the surfaces are covered by donor groups. N-CNTs have extra electrons due to the lone pair of electrons on nitrogen and P3HT contain conjugated π–electrons, which make them donor groups. The Gutmann acid (K_a_) and base (K_b_) constants were used to determine the surface chemistry of the samples. K_b_ values were higher for both N-CNTs and nanocomposites than K_a_ showing that the surfaces were covered by donor groups.

### 3.5. Photovoltaic Properties

**Figure 8:**
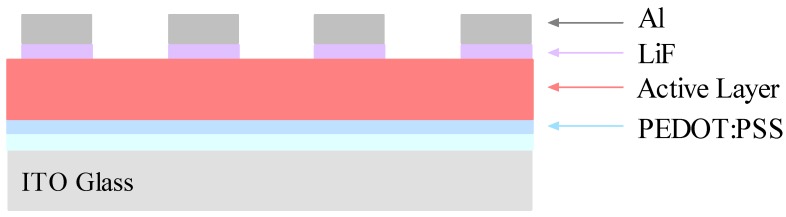
Diagram of the OSC showing the arrangements of the thin layers.

Several organic solar cells were fabricated by using a bulk heterojunction design in which the photoactive layer was composed of a blend of donor and acceptor molecules. Figure 8 shows a schematic diagram of the OSC device structure employed in this investigation. The electrical properties of the devices were studied by measuring the current-voltage (J-V) characteristics from each diode in the sample.

The important parameters of the cell are derived from the diode equation, which often describes the J-V characteristics of a diode. The fill factor (FF), which determines the quality of the device, and the power conversion efficiency (PCE), which provide the device output are defined as:

(3) $$FF=\frac{J_{Max}\times V_{Max}}{J_{sc}\times V_{oc}}$$

(4) $$PCE=FF\frac{J_{SC}\times V_{OC}}{P_{In}}$$

where J_MAX_ and V_MAX_ are current density and voltage at maximum power point; *J*_SC_ is short circuit current density; *V*_OC_ is open circuit voltage; and *P*_in_ is incident light power [35].

**Figure 9:**
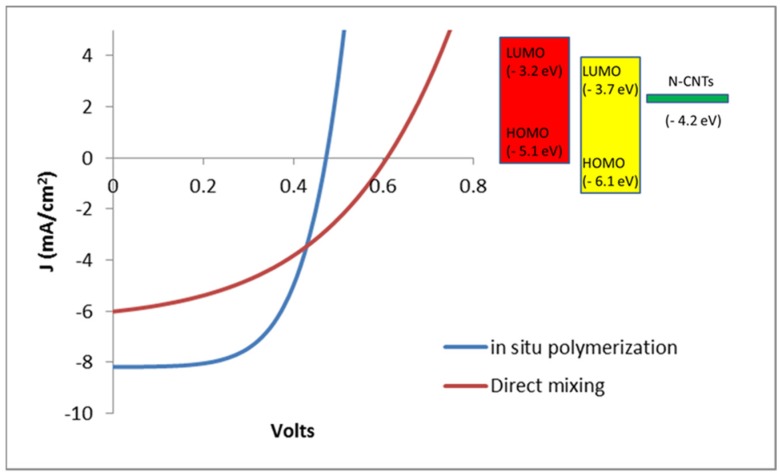
J-V curves of the devices from the two nanocomposites.

Characterization of the cell under light illumination was performed by using a solar simulator operating at AM 1.5, 100 mW·cm^−2^. The photoactive layers of the devices were fabricated from the two different nanocomposites obtained by *in situ* polymerization and direct solution mixing. Figure 9 shows the measured J-V curves of the devices produced under the two types of photoactive layers. The parameters of the solar cells derived from the data indicate that the devices prepared by *in situ* polymerization generally out performed those fabricated by direct solution mixing. According to the summary given in Table 2 the *J*_SC_, FF and PCE of the devices based on the nanocomposite synthesized by *in situ* polymerization are higher than those by direct mixing method.

The measured parameters of the cells are summarized in Table 2.

**Table 2:** Measured cell parameters.

Method of Synthesis	V_OC_ (volts)	Jsc (mA·cm^−2^)	FF	Efficiency (%)
Direct mixing	0.61	5.084	29.26	0.51
*In situ* polymerization	0.48	7.731	41.63	1.66
Reference device (P3HT:PCBM)	0.55	6.97	54.4	2.09

The higher J_SC_ suggests that better charge transport properties are exhibited in the photoactive layer prepared by *in situ* polymerization. In other words, the medium has better carrier mobility of charge carriers, which increases device performance. In fact, the SEM images of the two active layers given below partially explain difference in the devices performances. However, the open circuit voltage of the OSC prepared by direct solution mixing is higher than the former by nearly 135 mV, but, it is very close to the value of V_OC_ of the reference cell. This is an indication for the existence of high non-radiative recombination of the free charge carriers in the medium formed by *in situ* polymerization. Despite the higher solar cell performance of the *in situ* polymerization it suffers from high charge recombination process, which limits the potential ability of the medium for higher power conversion. The scanning electron microscopy (SEM) images were taken from various samples to investigate the morphologies of the active layers.

**Figure 10:**
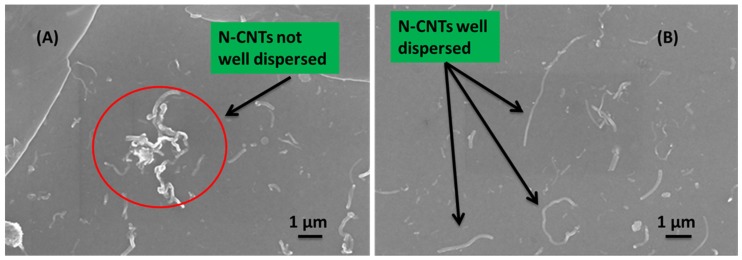
SEM images of the cell morphology showing N-CNTs (**A**) not well dispersed in the direct mixing nanocomposite; and (**B**) well dispersed for the *in situ* polymerization nanocomposite.

Figure 10 shows the surface morphologies captured from samples coated with the solutions of the active layer prepared both by direct solution mixing (Figure 10A) and *in situ* polymerization (Figure 10B). The SEM images clearly showed that the dispersion of the N-CNTs in the polymer matrix was not good for the nanocomposite formed by direct solution mixing. This composite favors CNT agglomeration and entanglement that form various CNT clusters Figure 10A. On the other hand, good dispersion of N-CNTs was observed for the nanocomposite prepared by *in situ* polymerization (Figure 10B) which can be ascribed to the fact that the monomers were polymerized on the surface of the N-CNTs and this inhibits π–π interaction of the tubes and thereby decrease agglomeration. Furthermore, good dispersion meant there was a continuous percolation path for free charge carriers and eventual collection at the electrodes thereby improving cell performance.

The high PCE and FF from devices prepared by *in situ* polymerization can be attributed to the small size of monomer molecules making the nanocomposite adduct more homogeneous compared with the one prepared by mixing solutions of polymer and N-CNTs [5]. Lee *et al.* [28] reported an efficiency of 3.8% for a nanocomposites of N-CNTs and P3HT prepared by direct mixing. The high efficiency of their device compared with ours could be due to preparation conditions. The nanocomposite in this work was prepared under ambient conditions whereas theirs was prepared in an inert gas atmosphere. Javier and Werner [32] used direct mixing to prepare a nanocomposite of pristine multi-wall CNTs (MWCNTs) and P3HT in ambient conditions. Their device recorded lower J_SC_ and FF than what we observed in our device from nanocomposites by direct solution mixing. We attribute the high J_SC_ and FF of our device to improved charge transfer by N-CNTs. Wu *et al.* [36] mixed pristine MWCNTs with P3HT and reported a high efficiency of 3.47% for devices prepared in an inert gas atmosphere.

From the above examples it is to be noted that most of the nanocomposites used in solar cells are more often prepared by direct solution mixing. However, according to the results found from the current synthesis and characterization, using FTIR, PL and J-V, it appeared to us that *in situ* polymerization would be the best technique for preparation nanocomposite. The poor performance of the devices prepared from direct solution mixing could be due to energetic agitation brought about by shear intensive mechanical stirring and ultrasonication used during nanocomposite preparation which initiates polymer chain breakage and degradation of opto-electrical properties [24].

## 4. Conclusions

*In situ* polymerization and direct solution mixing techniques have been used successfully to synthesize conducting nanonanocomposites of P3HT and N-CNTs. N-CNTs formed extra exciton dissociation sites which were observed by PL quenching. The diodes formed from the nanonanocomposites had positive rectification confirming their conductive nature but, the efficiency observed was very low. The *in situ* polymerization technique was observed to be better method for synthesising nanonanocomposites for organic solar cells. More investigation is required to determine why nitrogen-doping of the N-CNTs that is expected to improve the conductivity of the nanonanocomposites, did not improve the cell efficiency as anticipated.
